# Neuroinflammatory and Immunological Aspects of Fibromyalgia

**DOI:** 10.3390/brainsci15020206

**Published:** 2025-02-17

**Authors:** Kate Findeisen, Emma Guymer, Geoffrey Littlejohn

**Affiliations:** 1 Department of Rheumatology, Monash Health, Melbourne, VIC 3168, Australia; kate.findeisen@monashhealth.org (K.F.); geoff.littlejohn@monash.edu (G.L.); 2 Department of Medicine, Monash University, Melbourne, VIC 3800, Australia

**Keywords:** fibromyalgia, pain, neuroinflammation, immunonology

## Abstract

Fibromyalgia is a common, high-impact condition of chronic widespread pain and sensory dysfunction associated with altered central and peripheral sensory processing. A growing body of evidence supports the role of neuroinflammation and immune changes in fibromyalgia, and a narrative review of this literature was undertaken. Published data suggest that the interactions between the neural pain networks and the immune system in fibromyalgia appear to be bidirectional and operate both centrally and peripherally. There is a growing focus on processes occurring in the dorsal root ganglia and the role of maladaptive microglial cell activation. Ongoing insight into neuroinflammatory mechanisms in fibromyalgia opens potential avenues for the development of mechanism-based therapies in what is, at present, a challenging-to-manage condition.

## 1. Introduction

Fibromyalgia is a complex chronic condition characterised primarily by widespread musculoskeletal pain. Its prevalence in the general population ranges from 0.2% to 4.7%, with a higher incidence, particularly in middle-aged women, although the condition can arise at any age, including childhood [1]. Its clinical presentation is heterogeneous and fluctuating, with cardinal features including widespread musculoskeletal pain, fatigue, and sleep disturbance. Other manifestations include cognitive disturbance, mood disturbance and psychological distress, somatic and autonomic symptoms, and sensory and chemical sensitivity. Fibromyalgia is associated with various other clinical syndromes, including regional pain syndromes, particularly chronic back pain, headache and migraine, chronic fatigue syndrome, irritable bowel syndrome, and temporomandibular joint dysfunction [2]. The disease burden of fibromyalgia is significant, impacting an individual’s function and quality of life, with resultant socioeconomic impacts on health care systems and society [3,4].

Despite its prevalence and burden, the aetiology and pathophysiology of fibromyalgia remain incompletely understood, limiting the development of mechanism-based therapies. Two key mechanistic terminologies underpin our current understanding: nociplastic pain and central sensitisation. Nociplastic pain, distinct from neuropathic and nociceptive pain, is a third pain state in which pain arises from altered nociception despite no clear evidence of actual or threatened tissue damage [5]. It is believed to result from the augmentation of pain-related sensory pathways in the central nervous system (CNS), a process termed “central sensitisation”, characterised by alterations in pain processing in the CNS and dysfunction of descending inhibitory pathways [6]. Modulation of peripheral sensory pathways further contributes to increased sensitivity, alongside aberrations of other systems, including the autonomic nervous system and hypothalamic–pituitary–adrenal (HPA) axis [7].

**Figure 1:**
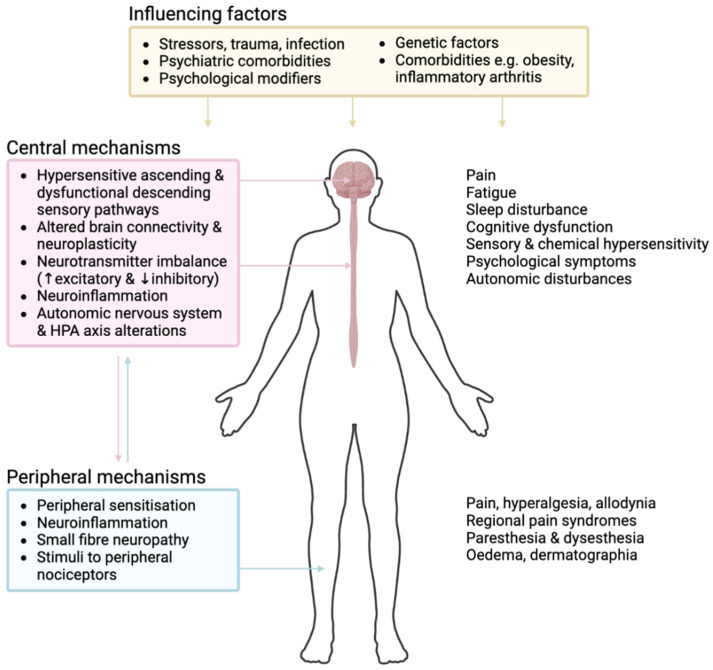
**Overview of pathogenic mechanisms in fibromyalgia.** Our understanding of the pathogenesis of fibromyalgia is evolving. Various predisposing factors may contribute to the development of fibromyalgia, where a complex interplay of multiple mechanisms occurs at both central and peripheral aspects of pain- and sensory-processing pathways, resulting in the sensitisation of ascending excitatory pathways and dysfunctional inhibitory pathways. Involved mechanisms include central neuroplasticity, neurotransmitter and neuropeptide imbalance, and modification and influence of the autonomic nervous system and HPA axis. Additional peripheral factors, such as ongoing nociceptive input and small fibre neuropathy, may also affect pathogenesis. Interaction between the nervous and immune systems, with resultant neurogenic neuroinflammation, contributes to many of these mechanisms. HPA, hypothalamic–pituitary–adrenal. Figure created with biorender.com.

The aetiology of fibromyalgia is best considered with a biopsychosocial model (Figure 1). Individuals with fibromyalgia have often experienced longstanding emotional, psychological and physical stress, including prior acute or chronic pain, trauma, abuse, and psychiatric disorders [8]. Strong associations exist with many types of chronic disease, particularly inflammatory rheumatic disease, as well as conditions such as non-genetic hypermobility [9,10], whilst various infections have been linked to the onset of fibromyalgia, including Epstein–Barr virus, Lyme disease, and COVID-19 [11,12,13]. Genetic factors are additionally involved, with increased rates of fibromyalgia and other chronic pain conditions in first-degree relatives of individuals with fibromyalgia [14]. Collectively, these predisposing factors result in background neural sensitivity, which is in turn compounded by cognitive–emotional distress and upregulated physiological stress responses, with resultant neuronal activation, blunting of descending sensory inhibitory systems, alterations in the HPA axis, and upregulation of the sympathetic nervous system. Whilst both “top-down” and “bottom-up” mechanisms have been conceptualised, it is likely that pain sensitisation results due to multidirectional interplay between multiple contributing pathways [15].

In recent years, there has been an increased focus on the role of neuroinflammation and immune system dysregulation in central sensitisation. Emerging evidence supports complex and bidirectional communication between the nervous and immune systems. Neuroinflammation is characterised by the activation of inflammatory cells, particularly microglia, and the release of pro-inflammatory molecules. These mediators interact with nociceptors and neurons in the peripheral and central nervous systems, altering their excitability and transmission, with the net result of augmented pain-processing pathways. This review aims to explore our current understanding of the pathophysiology of fibromyalgia with a focus on neuroinflammatory and immunological mechanisms.

This is a narrative review of the literature predominantly relating to published articles on fibromyalgia and neuroinflammation and immunology. Relevant papers were identified from searches on PubMed and online journals. The following keywords were used in various combinations to search PubMed: “fibromyalgia” AND each of “neuroinflammation”, “immune function”, or “inflammation”. Additional literature was identified by searching the reference lists of relevant identified papers and based on the authors’ own databases, and information from abstracts was included if deemed relevant. Criteria for selecting or excluding articles principally related to whether information within the identified study contributed to the theme of the narrative review, that is, the role of neuroinflammation in fibromyalgia.

## 2. Central Neuroinflammation

### 2.1. Neuroinflammation and Immune Changes in the Brain in Fibromyalgia

Studies utilising various brain imaging modalities provide increasing evidence for central sensitisation and its underlying mechanisms, including neuroinflammation. Functional magnetic resonance imaging studies of the brain confirm alterations in central networks involved in brain–spinal cord modulation, revealing increased connectivity between brain regions involved in the processing of pain and emotion and decreased connectivity with pain-inhibitory pathways [16,17]. Proton spectroscopy studies demonstrate alterations in specific neurotransmitters involved in pain and sensory transmission, with increased glutaminergic activity and decreased gamma-aminobutyric (GABA)-ergic activity, particularly in the posterior insula, which is responsible for modulating sensory input and neuroendocrine output [18,19,20].

The presence of microglial-mediated neuroinflammation is supported by positron emission tomography (PET) studies utilising radioligands that bind to the 18 kDa translocator protein (TSPO). Receptors of TSPO are upregulated on the mitochondrial membrane of activated microglial cells and thus can be used as a surrogate marker of neuroinflammation. [11C]PBR28 PET demonstrated increased radioligand binding in widespread cortical brain regions, including the superior parietal lobe, primary somatosensory cortex, motor cortex, supramarginal gyrus, prefrontal cortex, supplementary motor area, posterior cingulate, and precuneus, in individuals with fibromyalgia compared to healthy controls [21]. Uptake in the cingulate cortex was associated specifically with fatigue severity. Similar findings have been seen in a study utilising TSPO ligand [18F]DPA-714, which additionally distinguished high-affinity binders, demonstrating higher binding in the right post-central gyrus and right occipital and right temporal grey matter, regardless of genetically determined TSPO binding affinity, with additional regions demonstrating increased uptake in high-affinity binders [22]. Increased binding in the right parietal grey matter was associated with decreased quality of life, increased pain, and cognitive symptoms. [11C]-(R)-PK11195 PET studies also demonstrated increased microglial-mediated neuroinflammation in similar brain regions in fibromyalgia patients compared to healthy controls, as well as decreased neuroinflammation in fibromyalgia patients in the medulla, superior temporal gyrus, and left amygdala [23]. This study additionally identified differences between individuals with fibromyalgia versus complex regional pain syndrome (CRPS), with higher binding levels, particularly in the left pre- and post-central gyrus in the fibromyalgia group, and in the medulla, left insula, left thalamus, left superior temporal gyrus, putamen, and medial orbital gyri in the CRPS group. Correlations with certain regions and pain and stress scores were seen.

The role of central glial cell-mediated neuroinflammation is further supported by cerebrospinal fluid (CSF) studies, which demonstrate characteristic neuroinflammation patterns of increased interleukin (IL)-8 without consistent elevations in IL-1β in patients with fibromyalgia [24]. Endotoxin, a chemokine that prompts microglial release of pro-inflammatory cytokines, can be used to study neuroinflammatory responses. It can induce allodynia and hyperalgesia in healthy adults [25,26], and its administration activates brain microglia and increases inflammatory cytokines in [11C]PBR28-PET studies [27]. Magnetic resonance spectroscopic imaging has been used to further explore the link between chemokine-mediated immune system overactivation and central pain sensitisation. An abnormal thermal response after stimulation of immune response with endotoxin challenge was demonstrated in women with fibromyalgia, suggesting that fibromyalgia patients have abnormally sensitive central immune systems [28]. Taken together, these findings depict an integral role of microglia in CNS neuroinflammatory processes occurring in patients with fibromyalgia.

### 2.2. Neuroinflammation and Immune Changes in the Spinal Cord in Fibromyalgia

The dorsal horn has an established role in central sensitisation, with the first use of this terminology referring specifically to activity-dependent synaptic plasticity in dorsal horn neurons in response to nociceptor input [29]. It serves as the key relay centre for sensory information, particularly pain, with primary afferent neurons transmitting pain and other sensory information from the periphery synapsing at the dorsal horn. In central sensitisation, the dorsal horn becomes more sensitive to incoming stimuli via several mechanisms, including increased excitability of dorsal horn neurons via upregulation of receptors of neuroexcitatory peptides such as N-methyl-D-aspartate (NMDA) and decrease in inhibitory neurotransmitters such as GABA, resulting in amplification of a variety of sensory signals that then evoke pain responses [30,31].

Various mechanisms contribute to neuroinflammatory changes in the spinal cord. Neuropeptides and neurotransmitters, manufactured in the cell body of C fibres in the dorsal root ganglion, are transported distally to the periphery and proximally to the dorsal horn. These neuroactive chemicals act on neighbouring innate immune cells, particularly microglial cells, promoting their activation through the upregulation of Toll-like receptor 4 (TLR4). This in turn triggers the production and release of various excitatory substances and pro-inflammatory cytokines that act on local synapses to induce primary and remote hyperalgesia [32,33]. There is additional modulation of dorsal horn activity by descending corticospinal tracts, with attenuation of 5-hydroxytrpytaminergic-noradrenergic mechanisms from the midbrain and brainstem, resulting in impaired sensory inhibitory mechanisms and enhanced reactivity of sensory neurons to subthreshold and normal stimuli [7].

### 2.3. The Dorsal Root Ganglia in Fibromyalgia

Whilst technically part of the peripheral nervous system, there is growing evidence to suggest a key role of dorsal root ganglia (DRG) in fibromyalgia [34,35]. DRG house multiple inflammatory, immune, and nociceptive mediators. DRG neurons have unique physiological features enabling the generation of pain signals from varied afferent stressful stimuli, including psychological distress, which can induce DRG phenotypic change, resulting in hyperalgesia and chronic pain. Satellite glial cells (SGCs) closely envelop the cell bodies of sensory neurons in the DRG and are activated by nerve injury, inflammation, and stress, releasing pro-inflammatory and nociceptive molecules, which in turn activate nociceptive neurons [36]. DRG and SGC closely interact with the paravertebral sympathetic ganglia; SGCs express β2 adrenergic receptors, and DRG sympathetic sprouting prompts β2 adrenergic receptor overexpression [37]. Autoimmune mechanisms involving anti-SGC IgG antibodies have also been discovered [38,39]. These neuroinflammatory changes support a key role of the DRG in fibromyalgia pathogenesis.

## 3. Neuropeptides, Cytokines, and Chemokines

Altered levels of inflammatory and immunoregulatory cytokines in plasma and CSF have been demonstrated in fibromyalgia, supporting the role of immune dysregulation and neuroinflammation. Methodological comparison of data in this area is challenging; however, the most consistent cytokine profile involves increases in pro-inflammatory cytokines tumour necrosis factor (TNF)-α, IL-6, and IL-8 and the anti-inflammatory cytokine IL-10, alongside chemokine eotaxin [40]. The precise source and role of these cytokines in the development and maintenance of symptoms in fibromyalgia requires further study but is likely bidirectional. Immune system dysregulation results in alterations of pro-inflammatory mediators, which sensitise peripheral and central pain pathways, which activate the innate and adaptive immune system, in turn promoting the secretion of cytokines and other neuroinflammatory chemicals. Peripherally, inflammatory cytokines activate and sensitise nociceptors through upregulation of response to nitric oxide and prostaglandin E2, inducing pain and contributing to hyperalgesia. Centrally, pro-inflammatory mediators act on microglial cells and neurons to contribute to the above-described processes involved in central sensitisation [41,42].

There is an unclear correlation between increased inflammatory mediators and disease severity. One study demonstrated that plasma concentration of both IL-8 and monocyte chemoattractant protein-1 (MCP-1) correlated with pain severity, although not with overall fibromyalgia burden [43]. Increases in CSF concentrations of IL-8, but not IL-1β, are consistent with fibromyalgia being mediated by glial cell activation in the central nervous system rather than prostaglandin-related pathways [24]. The synthesis of IL-8 is dependent on sympathetic activation, potentially explaining the link between stress and central pain sensitisation [44,45]. Cytokines exert an effect on the sympathetic nervous system and HPA axis, which in turn contribute to top-down mechanisms that promote central sensitisation.

Neuropeptides are involved in multiple physiological processes, from neurotransmission to regulation of hormone function, immune response, and emotional regulation, and are present in the CNS, dorsal root ganglia, nerves, and peripheral tissues. Elevated levels of various neuropeptides that cause neuroinflammation are seen in the CSF and plasma of individuals with fibromyalgia. Substance P is the neuropeptide most consistently demonstrated to be elevated fibromyalgia [46,47], although other neuropeptides such as MCP-1, brain-derived neurotrophic factor, corticotrophin-releasing hormone (CRH), hemokinin-1 (HK-1), and calcitonin gene-related peptide are also implicated [48,49,50,51]. Substance P and other neuropeptides activate microglial cell release of pro-inflammatory mediators and other neuropeptides [52], further contributing to pain sensitisation and amplification.

## 4. Autoantibodies and Autoimmunity

A growing body of research supports the pathogenic role of anti-SGC IgG antibodies in fibromyalgia, highlighting the importance of the dorsal root ganglia in fibromyalgia pathogenesis and suggesting a potential role for autoimmune mechanisms. Anti-SGC IgG antibodies are elevated in the serum and plasma of individuals with fibromyalgia [38,53]. Elevated antibody levels correlate with disease severity, particularly higher self-reported pain, although not with other characteristics such as pain pressure threshold or disease duration [38,53]. Elevated antibody levels were not seen in all study patients, with large variations between individuals, suggesting that pathogenic autoantibodies may contribute to the pathogenesis of fibromyalgia in only a subset of patients [38]. Anti-SGC IgG antibodies are additionally elevated in painful post-COVID syndrome but not COVID-recovered controls [54], nor in other non-nociplastic chronic musculoskeletal pain conditions such as osteoarthritis [38].

Passive transfer of IgG from fibromyalgia patients to mice results in sensory hypersensitivity, with increased sensitivity to noxious mechanical and cold stimulation alongside increased nociceptor nerve fibre excitability [39]. Mice additionally demonstrated reduced locomotor activity, paw grip strength, and small fibre skin innervation. Passive transfer of IgG-depleted serum did not have the same effect. IgG from fibromyalgia patients was consistently detected in mouse DRG but not spinal cord or brain, with localisation of IgG primarily to satellite glial cells and fibre tracts entering the DRG, with evidence of increased activity of satellite glial cells [39]. Serum from fibromyalgia patients acutely activates murine DRG pro-nociceptive cells, with more intense and widespread stimulation compared to healthy controls [55]. A recent study demonstrated the binding of patient sera IgG autoantibodies to rat DRG neurons in 37% of patients with fibromyalgia but in none of the controls, with binding to SGC associated with symptom severity. This further supports the notion of distinct fibromyalgia subgroups with clinically relevant differences in immune-related pathogenic mechanisms [56].

Anti-SGC IgG antibodies have additional potential mechanistic roles in the brain, with a negative correlation between anti-SGC IgG and metabolite concentrations in the rostral anterior cingulate cortex and thalamus [53]. The potential contribution of antibodies to disease pathogenesis has been suggested in other nociplastic pain conditions, including CRPS [57,58].

## 5. Cellular Immune Aspects

**Figure 2:**
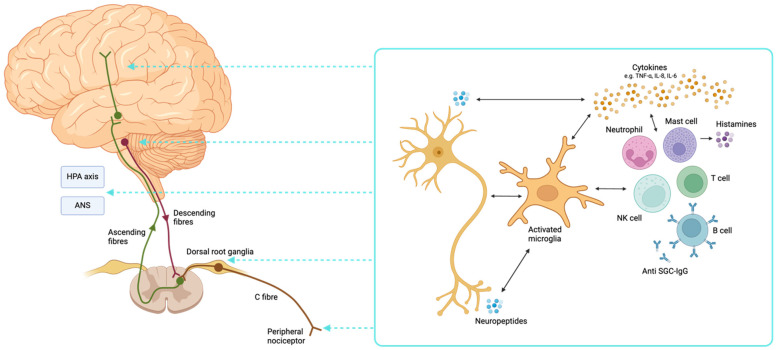
**Interactions between pain pathways and the immune system in fibromyalgia.** Neuroinflammatory mechanisms play a potential role in the development and persistence of fibromyalgia. A complex interplay between the nervous and immune systems exists both centrally and peripherally, with additional effects on endocrine and stress systems. Activated microglial cells play a key role in pro-inflammatory sensitising effects of the immune system on nociceptors and neurons, with bidirectional interactions with neuropeptides, cytokines, and various immune cells. Anti-SGC-IgG autoantibodies have also been demonstrated to potentially contribute to pathogenesis. HPA, hypothalamic–pituitary–adrenal; ANS, autonomic nervous system; TNF, tumour necrosis factor; IL, interleukin; NK, natural killer; SGC, satellite glial cell; IgG, immunoglobulin G. Figure created with biorender.com.

Cells of both the innate and adaptive immune systems play a role in neuroinflammation and central sensitisation and fibromyalgia (Figure 2). The most important cellular aspect is potentially the microglia, the macrophage of the CNS. Under normal conditions, these cells support neuronal function and maintain homeostasis; however, in response to noxious stimuli, injury, or stress, they become activated [59]. Microglia are capable of releasing either pro- or anti-inflammatory mediators via M1/M2 phenotypic switching and are essential for spinal and supraspinal pain processing. M1 macrophages produce pro-inflammatory mediators and induce nociceptive and nociplastic pain, whilst the anti-inflammatory response of M2 macrophages has inhibitory pain effects [60]. The unique plasma cytokine signature in fibromyalgia might stem from alterations in the M1/M2 ratio, favouring a pro-inflammatory state. The role of microglia in fibromyalgia is supported by imaging and cytokine studies, as outlined elsewhere in this review.

Other innate immune cells, including mast cells, neutrophils, and natural killer (NK) cells are potentially implicated in neuroinflammation and chronic pain. Mast cells release various neuro-sensitising inflammatory mediators, and their release is stimulated by various neuropeptides, including substance P [61]. The finding of elevated levels of these neuropeptides alongside IL-6 and TNF in fibromyalgia patients raises the possibility of mast cell origin of the inflammatory cytokine signature of fibromyalgia and supports a bidirectional interaction between pain-mediating substance P and mast cells [48]. Skin biopsies of individuals with fibromyalgia demonstrate a significantly increased number of mast cells in the papillary dermis compared to controls [62].

Neutrophils represent a key component of the innate immune system’s first line of defence. Their role in chronic pain is unclear but is potentially predominantly peripherally mediated as neutrophils are found in only small numbers in the CNS, typically only invading the blood–brain barrier in pathological states. Like other immune cells, neutrophils express opioid peptides on their surface. The precise role of immune-cell opioid peptides remains incompletely understood, with evidence to support the mediation of peripheral analgesia effect but also a contribution to hyperalgesia [63,64,65]. Neutrophils produce many of the cytokines identified to be altered in the plasma of fibromyalgia patients [40,66]. Despite their clear role in peripheral tissue damage, their involvement in neural pain pathways is less studied. One murine study demonstrated neutrophil infiltration of the dorsal root ganglia. Adoptive transfer of neutrophils from mice with chronic widespread pain or patients with fibromyalgia conferred mechanical hypersensitivity in recipient naive mice, suggesting a pronociceptive role of neutrophils [65]. Like other cellular aspects of the immune system, a bidirectional interaction between neutrophils and the nervous system is proposed [67].

The role of NK cells in fibromyalgia pathogenesis is also currently uncertain. NK cells are innate lymphoid cells with diverse functions, including regulation of immune responses, and contribute to the pathogenesis of immune-mediated diseases. NK cells also express Mu opioid receptors on their surface. Exposure to exogenous opioids has been associated with a reduction in the number and cytotoxic activity of NK cells [68,69], and this effect can be reverted by the opioid antagonist naltrexone [70]. Conversely, other studies have found no influence of opioid administration on NK cell activity and expression [71]. Skin biopsies of fibromyalgia patients reveal reduced NK cell expression of Mu receptors [72] and increased expression of NK cell activation ligands on subepidermal nerves with the presence of NK cells near peripheral nerves [73]. Changes in NK cell populations, such as depletion of CD56^bri^ NK cells and increased expression of adhesion molecules, have been demonstrated in fibromyalgia [73,74]. Depletion of circulating NK cells has been reported in other chronic pain conditions, suggesting that dysregulation of NK cells may contribute to chronic pain [75,76]. These studies suggest a potential role of NK cells in pain modulation, particularly peripherally.

Regarding the adaptive immune system, various studies have identified the possible role of T cells in fibromyalgia, although data are both inconsistent and inconclusive. The predominant finding is an alteration in subpopulations and function of T cells, particularly CD4+ T cells. Animal studies have linked CD4+ T cell depletion to inhibition of thermal hyperalgesia, tactile central sensitisation, and mechanical hypersensitivity following partial sciatic nerve ligation, alongside the reduction in stress-related behaviours [77,78]. Human studies in fibromyalgia have demonstrated increased Th1 type signature of CD4+ T cell subpopulations, with associated increases in interferon-gamma (IFNγ) and TNFα production and correlation with disease severity [79]. Interestingly, fibromyalgia patients treated with hyperbaric therapy demonstrated reductions in circulating Th1 cells, TNF-α, IFN-γ, and serotonin levels, and disease severity [79]. Interaction between the adaptive immune system and serotonin, a neurotransmitter involved in nociception, is further supported by the association of serotonin transporter 5-HTTLPR mutations and increased T cell activation in fibromyalgia patients [80]. Another study found reductions in cytotoxic CD8+ T cells in fibromyalgia and CRPS patients that correlated with post-traumatic stress scores without alterations in CD4/CD8 ratio [81], again suggesting a connection between pain, stress, and lymphocyte populations. Whilst the data support the hypothesis that T cell-mediated mechanisms may contribute to pain sensitisation, a causal relationship and its direction between T cell changes and pain pathogenesis is yet to be established.

Outside of the potential contribution of anti-SGC autoantibodies, there is a need for further study to support a major role of B cell-mediated autoimmune mechanisms in fibromyalgia. It is plausible that other unidentified autoantibodies are implicated. CD20-positive B cell antibodies have been linked to CRPS changes in murine models, with CD20 depletion reversing nociceptive sensitisation in mice with established CRPS-like changes [58]. Additionally, fibromyalgia is associated with changes in the gene-expression profile of peripheral B cells with increases in expression of interferon-related genes [82].

## 6. Peripheral Neuroinflammation

Whilst substantial evidence supports a primary role of central sensitisation in fibromyalgia, several important peripheral mechanisms exist, including peripheral neurogenic inflammation. Key to neurogenic inflammation is the Lewis triple response, whereby mechanical, thermal, or chemical stimulation of the skin results in a weal and flare response [83]. This response is produced by the release of pro-inflammatory peptides from the peripheral nerve ending of C fibres and is increased in patients with fibromyalgia [84]. The extent of flare response correlates with the severity of allodynia, reduced pain threshold, slow-wave sleep deprivation, and increased fatigue [84,85]. In addition to the Lewis response, peripheral neurogenic inflammation results in other clinical findings. Local soft tissue swelling and fluid retention are described, as is livedo reticularis [41]. Fibromyalgia patients who experience Raynaud phenomenon and/or livedo reticularis have significantly elevated levels of fibronectin, a tissue marker of endothelial activation [86]. The clinical findings of neurogenic inflammation are particularly pronounced in complex regional pain syndrome, suggesting differences in the contribution of peripheral neurogenic inflammation to the clinical features of these two conditions [41].

Peripheral neuroinflammation involves interactions between peripheral nociceptors, the innate and adaptive immune systems, and local tissue. Upon activation via axonal or dorsal root reflexes, C-fibre nerve terminals release a range of pro-inflammatory neuropeptides, particularly substance P, CGRP, and neurokinin A, which act on surrounding blood vessels, resulting in vasodilatation, increased vascular permeability, and neutrophil migration to local tissues [41]. CGRP acts on vascular smooth muscle and endothelial cells and is the key mediator of neurogenic vasodilatation, whilst substance P and neurokinin A increase vascular permeability via action on neurokinin A1 receptors and additionally directly recruit innate and adaptive immune cells [87]. Mast cells, found in an increased number of the skin of fibromyalgia patients, degranulate, releasing various pro-inflammatory, neurostimulatory, and vasoactive mediators, including but not limited to histamine, bradykinin, prostaglandins, TNF, serotonin, and endothelial growth factors [62,88]. These substances may in turn sensitise neighbouring nociceptive terminals and primary afferent neurons, further amplifying local inflammatory changes at the affected site. The release of pro-inflammatory cytokines from mast and other innate immune cells has a direct effect on nitric oxide and prostaglandin E, established mediators of inflammation [41]. T lymphocytes are additionally activated, also contributing to the pro-inflammatory cytokine profile described in fibromyalgia cohorts.

Local neuroanatomical changes in the morphology, neurophysiology, and function of peripheral nociceptors are described in fibromyalgia [89]. This is commonly referred to as small fibre pathology and is seen in approximately 50% of fibromyalgia patients, but also in neuropathic pain conditions [90,91]. Small fibre neuropathy selectively affects unmyelinated C and thinly myelinated Aδ fibres that are responsible for mediating pain and thermal sensation. Damage to these fibres results in sensory changes such as allodynia, hyperalgesia, paraesthesia, and dysesthesia, alongside autonomic dysfunction [92]. In fibromyalgia, there can be a reduction in the absolute number and density of non-myelinated nerve fibres in the skin, a reduction in mean axon diameter, and C-fibre structural change [89,93,94]. There continues to be debate on the role of small fibre neuropathy in fibromyalgia, and whilst it can be seen in association with central sensitisation, isolated small fibre neuropathy should be distinguished from the small fibre pathology seen in fibromyalgia [90]. One study found that the presence or absence of small fibre pathology in patients with fibromyalgia did not influence the sensory phenotype, with normal sensory phenotype compared to thermal and mechanical hyperalgesia being similarly represented [95]. This differed from those with isolated small fibre neuropathy, where the predominant phenotype was sensory loss and mechanical hyperalgesia. Overall, whilst half of the patients with fibromyalgia will have evidence of small fibre pathology, its relationship to the condition’s pathogenesis and symptomatology remains unclear, emphasising the current limitations of identifying and directing treatment at small fibre neuropathy in fibromyalgia.

## 7. Stress and Neuroinflammation

Fibromyalgia is considered a stress-related disorder. Stress is linked to both the development of fibromyalgia and related syndromes involving central sensitisation and symptom exacerbation [8,96]. This has led to our understanding that alterations in the sympathetic nervous system and HPA axis contribute to the pathogenesis of fibromyalgia, acting as an upstream generator and modulator of neuroinflammation. Patients with fibromyalgia demonstrate sympathetic hyperactivity, with individuals demonstrating decreased heart rate variability and blunted autonomic response to stress, and heart rate variability correlates with various symptoms, including pain severity [97,98]. Noradrenaline injections have also been shown to evoke significantly more pain in fibromyalgia compared to patients with rheumatoid arthritis or healthy controls [99]. The sympathetic nervous system can modulate peripheral nociceptor neurons as well as cells of the innate immune system through upregulation of alpha-1 adrenoceptors, contributing to neurogenic inflammation. Whilst sympathetic hyperactivity likely contributes to the development and maintenance of central and peripheral neuroinflammation, the direct causality of this relationship is less clear. Increased sympathetic activity is only seen in a portion of patients with fibromyalgia and appears to partially correlate with other comorbidities such as deconditioning and childhood trauma [98].

The gastrointestinal system potentially links stress and CNS neuroinflammation via other mechanisms, with growing interest in the gut–brain axis. Experimental models of depression demonstrate that chronic mild stress compromises the intestinal barrier, resulting in increased gastrointestinal permeability and translocation of lipopolysaccharides, which in turn contributes to pro-inflammatory mediators in the CNS via activation of TLR4 [100]. The gut microbiome has complex interactions with the immune, nervous, and neuroendocrine systems, representing a link between stress-related conditions and neuroinflammation [101]. Recent studies have investigated the role of the gut microbiome, specifically in fibromyalgia, observing certain alterations of gut bacteria, particularly in species involved in short-chain fatty acid and bile acid metabolism, as well as those known to have roles in modulation of immune function [102]. One rodent study demonstrated a causal role of the gut microbiome in fibromyalgia; however, the causal relationship between the gut microbiome and fibromyalgia needs further exploration [103].

The HPA axis and vagus nerve are additional key components of the stress-inflammation axis. Neurotransmitters demonstrated to be imbalanced in fibromyalgia influence both the sympathetic nervous system and HPA axis [104]. Study of the HPA axis is inconsistent, with evidence of both hyper- and hypoactivity, likely a result of chronic stress producing high basal tone and hyperactive stress responses but resultant hyporeactive HPA axis [105]. Alterations of the autonomic and HPA systems result in prefrontal cortex hypoactivity and amygdala hyperactivity, leading to dysfunction of vagal tone, although the directionality of this relationship is less clear [105,106]. Low vagal tone and sympathetic hyperactivity, as demonstrated by low heart rate variability, are seen in fibromyalgia [107], and chronic stress is also known to reduce vagal tone [108]. The vagus nerve is a key component of the neuroimmune axis and has anti-inflammatory properties via interactions with inflammatory mediators and vagal afferent and efferent pathways [105]. Afferent fibres, responsible for somatic, taste, and visceral sensation, may be activated by IL-1β, in turn activating the HPA axis with downstream inhibition of peripheral inflammation [105,109]. Efferent fibres represent the parasympathetic branch of the autonomic system and are also activated by cytokine-stimulated afferents in a self-reflex circuit that suppresses the production of cytokines [105,110]. Dysregulation of the circadian variation in cortisol and response to corticotropin-releasing hormone has been observed in fibromyalgia, alongside potential correlation with symptom severity, further implicating the HPA axis, although data in this area are inconclusive [111,112].

## 8. Therapeutic Implications

The multiple components involved in the pathogenesis and persistence of fibromyalgia necessitate a multimodal approach to treatment. Current treatment relies particularly on non-pharmacological strategies with modest efficacy of available pharmacotherapies, leaving an area of significant unmet clinical need. Neuroinflammation compromises a complex set of interacting elements with multiple potential therapeutic targets. Various targets have already been explored, with many dispelled as ineffective in fibromyalgia management. Neither non-steroidal anti-inflammatory drugs nor glucocorticoids appear to suppress neuroinflammation or improve the symptoms of fibromyalgia [113,114]. Biologic drugs targeting inflammatory cytokines, including those used in other autoimmune and inflammatory conditions, have not been studied in a randomised controlled fashion in fibromyalgia, and there are insufficient data to support their efficacy [115]. In patients with rheumatoid arthritis, there are data to suggest that JAK inhibition significantly improves pain outcomes compared to available biologic disease-modifying anti-rheumatic drugs (DMARDs), whilst IL-6 inhibition may reduce disproportionate articular pain in rheumatoid arthritis patients [116,117]. Both studies explore pain in rheumatoid arthritis as opposed to concomitant fibromyalgia; there are no current data to support the use of these therapies in fibromyalgia alone. However, it raises the possibility that biologic and targeted synthetic DMARDs may have analgesic effects outside of their disease-modifying mechanisms, potentially via neuroinflammatory pathways mediating peripheral and central sensitisation.

Several existing therapies target central sensitisation and neuroinflammatory mechanisms. Naltrexone, an μ opioid receptor antagonist that can cross the blood–brain barrier, suppresses the pro-inflammatory action of activated microglia. At low doses, naltrexone increases TLR4, thereby modulating M1/M2 switching in favour of anti-inflammatory M2 phenotype [118]. Serotonin and norepinephrine reuptake inhibitors are recommended treatment options in fibromyalgia, and there is evidence to suggest reciprocal pathways linking serotonin to inflammation and immune activation [119]. The dual reuptake inhibitor milnacipran reduces ventricular lactate levels, a possible marker of central neuroinflammation [120]. Tricyclic antidepressants are prescribed for a wide range of neuroinflammatory disorders, including fibromyalgia, with evidence to support their modulation of the neuroimmune interface, including effects on glial cells, T cells, and pro-inflammatory cytokines [121,122,123]. Gabapentinoids also act upon neuroinflammatory pain pathways, attenuating glial cell activation and suppressing lipopolysaccharide-induced IL-6 production in the spinal dorsal horn, alongside more direct action on the neurotransmitter GABA [124].

Non-pharmacological aspects of fibromyalgia management also induce a neuroimmunomodulatory response. Modification of stress via education, exercise, and psychological strategies can modulate stress responses that drive neuroinflammation [41]. Mindfulness-based stress reduction reduces stress and can decrease cutaneous neurogenic inflammatory responses and modify pro-inflammatory cytokine profiles [125,126]. Exercise-based interventions were shown to reduce pro-inflammatory biomarkers, particularly IL-8 [127]. Addressing other lifestyle factors, including those with recognised links to systemic inflammation and neuroinflammation, such as diet, obesity, and gastrointestinal dysbiosis, may have additional impacts on peripheral and central neuroinflammation [128,129]. The Mediterranean diet, believed to have anti-inflammatory effects, has been found to improve fibromyalgia parameters, including fatigue, disability, and anxiety [130]. Taken together, these studies indicated that non-pharmacological intervention has a potentially powerful impact on the symptomatology of fibromyalgia.

The study of anti-satellite glial cell (SGC) IgG antibodies raises the possible efficacy of IgG-reducing therapies in fibromyalgia. A clinical trial of rozanolixizumab, a humanised IgG4 monoclonal antibody that binds to and reduces circulating IgG, is currently underway in patients with severe fibromyalgia. This aims to assess whether the reduction in IgG antibodies will improve symptom severity [131]. Other techniques targeting glial cell activation may offer therapeutic promise. Ongoing evolution of our understanding of the role of neuroinflammation in fibromyalgia will hopefully identify other potential treatment targets.

## 9. Limitations

Due to the narrative nature of our review, there remains the possibility that there may be some bias in the data represented. Published studies in this field are frequently small in number, differ in methodology, and are not always characteristic of clinical groups. As such, judgement is exercised when including information in the review, including the relevance of preliminary studies. All these factors contribute to the potential limitations of our review.

## 10. Conclusions

Neuroinflammatory mechanisms play a pivotal role in the development and persistence of the clinical features of fibromyalgia. A complex interplay between the nervous and immune systems exists both centrally and peripherally, with additional effects on endocrine and stress systems. Improved understanding of the causality and direction of these processes is required to determine if neuroinflammation is a primary pathophysiological mechanism as opposed to a secondary phenomenon of stress. Additionally, future experimental study focusing on the diversity of fibromyalgia and the identification of clinical subgroups in which immune mechanisms are implicated is required. This will aid further exploration of the therapeutic role of targeting specific components of neuroinflammation.

## Data Availability

No new data were created or analysed in this study. Data sharing is not applicable to this article.
